# The interaction of Glu294 at the subunit interface is important for the activity and stability of goose δ-crystallin

**Published:** 2009-11-14

**Authors:** Chih-Wei Huang, Yu-Hou Chen, Ya-Huei Chen, Yun-Chi Tsai, Hwei-Jen Lee

**Affiliations:** 1 Department of Biochemistry, National Defense Medical Center, Taipei, Taiwan; 2 Department of Pharmacy Practice, Tri-Service General Hospital, Taipei, Taiwan; 3 Genomics Research Center, Academia Sinica, Taipei, Taiwan

## Abstract

**Purpose:**

δ-Crystallin is a soluble structural protein in found in avian eye lenses; it shares high amino acid sequence identity with argininosuccinate lyase. E294 is the only residue located at the double dimer interface and it performs hydrogen bonding with the active site residues of H160 and K323 in the neighboring and diagonal subunits, respectively. H160 is reported to play an important role in catalysis due to its H-bond interaction with the fumarate moiety of the substrate. In order to clarify the function of E294 in either stabilization of the quaternary structure or in catalysis, we carried out site-directed mutagenesis and functional analysis.

**Methods:**

The structure of both wild-type and mutant proteins were analyzed by circular dichroism (CD) spectroscopy, fluorescence spectra, and analytical ultracentrifugation. Structural stability was measured by CD and tryptophan fluorescence. A modeled structure of the E294L mutant was built and optimized with energy minimization.

**Results:**

No gross structural changes were observed when E294 was substituted with leucine, as judged by circular dichroism, tryptophan fluorescence, ANS fluorescence, and sedimentation velocity analyses. However, this mutant enzyme had only about 10% of the activity of a wild-type enzyme and its secondary structure was more easily denatured by increased temperature than that of a wild-type enzyme. The mutant protein also underwent its first unfolding transition at a lower concentration of guanidinium-hydrochloride than the wild-type protein.

**Conclusions:**

These results indicate that the interactions offered by E294 in the dimer-dimer interface of δ-crystallin are required to maintain the hydrogen bonding network in the active site for catalysis. Disruption of the interaction had no significant effect on the conformation and quaternary structure of δ-crystallin but it did lead to instability in the double dimer structure.

## Introduction

δ-Crystallin is the main soluble protein found in the eye lenses of reptiles and birds and it confers  special refractive properties. This taxon-specific crystallin was recruited from a metabolic enzyme, argininosuccinate lyase (ASL), by a process known as gene sharing [1-5]. ASL catalyzes the reversible breakdown of argininosuccinate into arginine and fumarate, a reaction involved in arginine biosynthesis [6]. The two proteins share about 70% sequence identity and similar overall topology [2,7-9]. Gene duplication has enabled two isomeric proteins, δ1- and δ2-crystallin, to be identified. They share over 90% identity in amino acid sequence but only δ2-crystallin retains enzyme activity [10].

**Figure 1:**
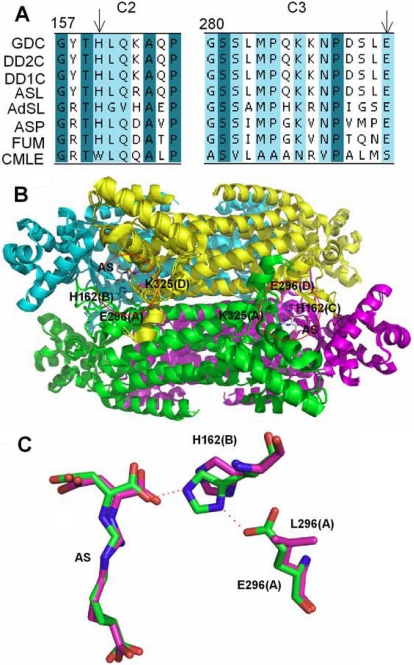
Comparison of sequence and structure of δ–crystallin and the ASL super-family. **A**: Conserved regions of c2 (157–166) and c3 (280–294) are derived from multiple-sequence alignment using the program ClustalW [30]. Amino acid sequences that are identical or highly conservative are shaded in dark green and cyan, respectively. Abbreviations used: GDC, goose δ–crystallin; DD2C, duck δ2–crystallin; DD1C, duck δ1–crystallin; ASL, argininosuccinate lyase; FUM, fumarase; ASP, aspartase; CMLC, 3-carboxy-*cis*-*cis*-muconate lactonizing enzyme. H160 and E294 are indicated by arrows. **B**: Superimposition of the quaternary structure of goose δ–crystallin and duck T161D δ2–crystallin (PDB accession no: 1XWO and 1TJW). Monomer A, B, C and D are shown as a cartoon and colored green, cyan, magenta and yellow, respectively. The red circle depicts the active site. Related residues are highlighted as sticks. Argininosuccinate (AS) is shown as stick and carbon atoms are colored grey. **C**: Superimposition of residues in substrate binding site of duck T161D δ2–crystallin structure and T161D/E296L δ2–crystallin model. E296, L296, H162 and AS are displayed as stick models and shown in green and magenta for T161D and T161D/E296L proteins, respectively. Interactions between E296 and H162, and H162 and AS are highlighted by dashed orange lines. The parentheses after each residue represent the defined subunit.

δ-crystallin and ASL belong to a super-family of metabolic enzymes which act as homo-tetramers. Members of this super-family, including class II fumarase [11], aspartase [12], adenylosuccinate lyase [13], and 3-carboxy-*cis*-*cis*-muconate lactonizing (CMLE) enzyme [14], share low-sequence homology but have three highly conservative sequence regions (Figure 1A). Regions from the different subunits are assembled to form the active site of the functional enzyme.

δ-crystallin has a double dimer quaternary structure [9,15,16]; each of its monomers has three domains, with domain 2 containing five helices that associate with other monomers to form the core structure of the protein. Hydrophobic interaction is the major force for subunit assembly, in addition to hydrogen bonds and salt bridges. Dissociation of δ-crystallin occurs in the presence of guanidinium-hydrochloride (Gdm-HCl) leading to the formation of a monomeric intermediate. Dimers are transiently observed in the unfolding process [17,18].

Truncation of the NH_2_-terminus, which interacts with a hydrophobic cavity in a neighboring monomer, results in disassembly of the double-dimeric structure [15]. Disruption of inter-facial interactions provided by the side-chains of K315 and E327 in domain 2 also led to dissociation of double dimers [19]. These results indicate the importance of these interactions in stabilizing the quaternary structure of δ-crystallin. In the present study proposes to investigate the role of E294. This residue is the only residue located at the dimer-dimer interface; it forms hydrogen bonds with active site residues H160 and K323 when the three residues are brought together from different monomers through subunit association (Figure 1). E294 and H160 are located in the active site and are highly conserved throughout the whole super-family (Figure 1). The structure of duck T161D δ2-crystallin in complex with argininosuccinate shows that H162 (which is equivalent to H160 in goose δ-crystallin) interacts directly with the fumarate moiety of argininosuccinate by hydrogen bonding and is assumed to function as a general base for catalysis [20,21]. In our study, site-directed mutagenesis was performed and biophysical analysis revealed the important role of E294 in catalysis. Disruption of the interaction had no effect on the conformation and quaternary structure of δ-crystallin but led to instability of the double dimer structure of δ-crystallin.

## Methods

### Materials

All chemicals were of analytic grade or higher and were purchased from Sigma-Aldrich (St. Louis, MO) or J.T. Baker (Phillipsburg, NJ). Restriction enzymes and T4 DNA ligase were purchased from New England Biolabs (Beverly, MA). Chromatography systems and columns were supplied by GE Healthcare (Uppsala, Sweden).

### Preparation of mutant

The E294L mutant construct was generated by PCR amplification using the Stratagene QuikChange mutagenesis system (Stratagene, La Jolla, CA) with a pET-gδ template [15] and the following forward and reverse primers: 5'-CCT GAT AGC CTG CTA CTG ATC CGC AGC-3' (forward) and 5'-GCT GCG GAT CAG TAG CAG GCT ATC AGG-3' (reverse). After amplification, template DNA was digested with DpnI and newly-synthesized mutant-containing vectors were transformed into *E. coli* DH5α competent cells. The complete DNA sequences of the mutant plasmids were determined.

### Protein expression and purification

Cultures containing the recombinant plasmid were fermented and crude extracts were prepared as reported previously [15]. The supernatants were loaded onto a Q-Sepharose anion exchange column (HiPrep 16/10 Q XL), pre-equilibrated in buffer A (50 mM Tris-HCl buffer, pH 7.5) and eluted with a linear gradient of 0 to 0.4 M NaCl. A 40%–55% ammonium sulfate fractionation was performed on the pooled fractions. The pellets were collected and dissolved in 5 mL of buffer A and loaded onto an S-300 Sephacryl column (26 mm x 85 cm) pre-equilibrated in buffer A. Fractions were analyzed by SDS-PAGE and Bradford analysis.

Proteins possessing COOH-terminal His_6_ tags were purified using a metal-chelating column (Chelating Sepharose FF) charged with Ni^2+^ and equilibrated in 50 mM Tris-HCl buffer, pH 7.5 and 0.5 M NaCl (buffer A). Unwanted bound protein was eluted with buffer A containing 60 mM of imidazole-HCl. δ-Crystallin was eluted using 0.5 M of imidazole-HCl in buffer A. The purified protein was exchanged into 50 mM Tris-HCl buffer, pH 7.5, using a Sephadex G-25 column (26 mm×12 cm).

### Enzymatic activity assay

The argininosuccinate lyase activity of the δ-crystallin was measured by monitoring the absorption of fumarate at 240 nm in a Perkin-Elmer Lambda 40 spectrophotometer. Assays were performed in 50 mM Tris-HCl buffer (pH 7.5) with 1 mM sodium argininosuccinate as the substrate. A molar absorption coefficient of 2.44×10^3^ M^-1^cm^-1^ was used for all calculations [22].

### Fluorescence studies

The fluorescence spectra of δ-crystallin were measured using a Perkin-Elmer LS-50 luminescence spectrophotometer equipped with a thermostatically-controlled sample holder. δ-crystallin with various concentrations of Gdm-HCl in 50 mM Tris-HCl buffer, pH 7.5 was incubated at 25 ^o^C overnight. Intrinsic tryptophan fluorescence spectra of the protein were recorded with excitation wavelength set at 295 nm and bandwidths set at 5 nm for both excitation and emission wavelengths. All spectra were corrected for buffer or denaturant absorption. The average emission wavelength (which registered variation in both redshift and fluorescence intensity) was recorded for the purpose of data analysis [23]. The unfolding curve as a function of Gdm-HCl concentration was globally-fitted to a four-state transition model [15].

δ-Crystallin (0.1 mg/ml) with 0.2 mM ANS (1-anilinonaphthalene-8-sulfonic acid; Molecular Probes, Eugene, OR ) was incubated in the dark for 20 min. The fluorescence spectrum of ANS was recorded from 450 to 550 nm with the excitation wavelength set at 370 nm. The bandwidths were set at 5 and 10 nm for excitation and emission wavelength, respectively.

### Circular dichroism studies

Circular dichroism (CD) spectra were obtained using a Jasco J-810 spectropolarimeter (Jasco, Tokyo, Japan) with a thermostatically-controlled sample holder. Experiments were performed in 50 mM Tris-HCl buffer (pH 7.5) using a 1 mm path-length cell for the far-UV region (200 to 250 nm). All spectra were averaged from three accumulations and were buffer-corrected. The mean ellipticity of the residue [θ] was calculated [24]. The thermostability of secondary structures was monitored by recording ellipticity at 222 nm in a circulating bath with a programmable temperature control (Neslab, Thermo Fisher Scientific, Waltham, MA) at a scan rate of 1.0 ^o^C per min.

### Analytical ultracentrifugation studies

Sedimentation of the proteins was analyzed using a Beckman-Coulter (Palo Alto, CA) XL-A analytical ultracentrifuge (AUC) with an An50 Ti rotor. Sedimentation was performed at 20 ^o^C and 130,000× g in standard double-sector aluminum centerpieces. The radial scans were recorded at 5-min intervals for about 3 h. SETFIT software was used for data analysis [25].

### Construction of mutant enzyme model

The E294L mutant δ-crystallin was constructed with Discovery Studio software, using the “Build mutants” protocol from the “Protein modeling” module (Accelrys Discovery Studio 2.0; Accelrys Software Inc., San Diego, CA). The structure of the duck T161D mutant δ-crystallin in complex with argininosuccinate (PDB access no: 1TJW) [20] was used as a template for building the E296L mutant model (equivalent to E294 in goose δ-crystallin). Energy minimization for optimization of residue geometry was applied to the T161D/E296L mutant model using the “minimization” protocol of the “simulation” module; this involved applying the algorithm of smart minimization until the gradient tolerance was satisfied (RMS Gradient ~ 0.1 kcal/mol/Å). Binding energy for the argininosuccinate was calculated using “Calculate binding energy” from the “Receptor-ligand interactions” module.

## Results and Discussion

Goose δ–crystallin has a double dimer quaternary structure. The proper assembly of two pairs of dimers is critical for a functional protein. E294 is a residue that provides interactions between each of the subunit interfaces. The OE2 atom in the carboxyl group of E294 is able to form hydrogen bonds with different residues in the diagonal and neighboring monomer at the dimer-dimer interface: it interacts with the imidazole group of H160 and with the amino group of K323 (Figure 1B).

Side-directed mutagenesis was used to investigate the function of E294. Sequencing confirmed that only the desired mutation had been introduced into the enzyme. Circular dichroism analysis suggested the secondary structure of the E294L mutant was highly similar to that of the wild-type protein, with fractional α-helical content of about 0.64±0.02 (E294L) and 0.66±0.01 (wild-type). The fluorescence spectrum of tryptophan residues and ANS for wild-type enzyme and the E294L mutant were also highly similar, suggesting the conformation of the mutant enzyme greatly resembled that of the wild-type enzyme. Sedimentation velocity analysis for E294L revealed a single component with a molar mass distribution profile almost overlapping that of the wild-type protein. The estimated sedimentation coefficient and molecular mass were 9S and 190 kDa, respectively, indicating that the E294L mutant had retained its tetramer structure. These results suggest that the inter-subunit interactions provided by this residue might not play a large role in the assembly of protein quaternary structure.

The catalytic properties of the mutant enzyme were then investigated. The specific activity of the mutant enzyme was only about 10% of the wild-type enzyme (Table 1). H160 and E294 are highly conservative residues across the ASL/fumarase super-family (Figure 1A). In the structure of duck T161D δ2-crystallin these two residues are located in the active site and form a hydrogen-bonding network with the fumarate moiety of argininosuccinate which is converted into fumarate (Figure 1B). The rms deviation of the C_α_ atom between the structure of the goose δ-crystallin and duck T161D δ2-crystallin was about 0.49 Å. Because of the small difference between these two structures, the structure of duck T161D δ2-crystallin (in complex with argininosuccinate) was used as a template for modeling the structure of the E296L mutant (E296 is equivalent to E294 in goose δ-crystallin). Comparing the modeled structure of the E296L mutant with the structure of duck T161D δ2-crystallin showed that, without the carboxyl group found in E296, the E296L mutant displays a 1.5 Å shift of the imidazole side-chain of H162 (colored magenta in Figure 1C). This movement destroys the hydrogen bonding between the ND1 atom of H162 and the O_γ_2 atom of the fumarate moiety of argininosuccinate, which in turn creates a 0.8 Å shift of the fumarate moiety. The calculated binding energy for argininosuccinate in the modeled E296L mutant structure was about 117 kcal/mol, less than that calculated for the duck T161D δ2-crystallin (Table 1). These results demonstrate the critical role of E296, which stabilizes the H162 residue in a proper conformation for interaction with the substrate. Furthermore, E296 was assumed to participate in a charge-relay system with H162 [21]. Previous mutation studies have suggested that H162 is involved, directly or via a water molecule, in abstracting a proton from the C_β_ atom of the fumarate moiety [9,21,26,27]. Inactivation of the E296L mutant is consistent with its modeling structure in which hydrogen bonding between these two residues does not occur.

**Table 1:** Summary of parameters for wild-type and mutant δ-crystallin

**Proteins**	**SA (U/mg)**	**CBE (kcal/mol)**	**Tm (°C)**
WT	16±4.0	-697±1	70±0.1
E294L	1.7±0.3	-580±6	65±0.5

**Figure 2:**
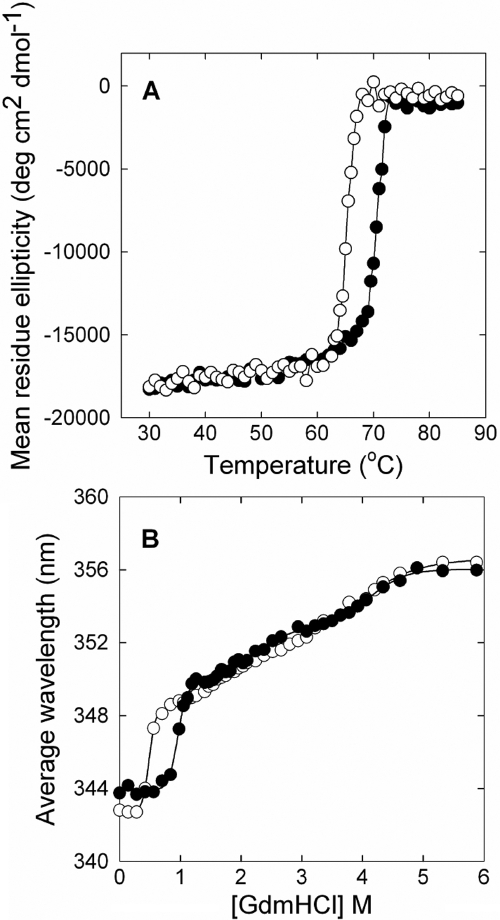
Structural stability of δ-crystallin. The labels represent the protein samples of wild-type (○) and E294L (●). **A**: Thermostability. The ellipticity at 222 nm was monitored at various temperatures. **B**: Stability in the presence of Gdm-HCl. Tryptophan fluorescence was measured in response to various concentrations of Gdm-HCl. The solid lines were obtained from the result of global fitting to the equation based on a 4-state model [15]. The protein concentrations used in the assays were 0.5 mg/ml for (**A**) and 0.03 mg/ml for (**B**).

SA represents specific activity in nmol/min/mg. *T*_m_ is derived from CD data in Figure 2A. CBE is the calculated binding energy for argininosuccinate derived from computation using module as described in Materials and Methods.

The application of protein structure to elucidate the results of functional analysis has also been reported previously with mutant duck δ2-crystallin. One example looks at the T161S and T161D structures [20]. In the active site, the hydroxyl group of Ser161 interacts with Lys289 via a water molecule in a similar orientation as Thr16, supporting the ~70% activity retention of the mutant enzyme. In contrast, the small conformational changes in the side-chain of Asp161, together with reorientation of the substrate, may provide an explanation for the loss of activity in the T161D mutant protein. These results from other structural and biochemical studies suggest that a hydroxyl group is required at position 162 to help correctly position the side-chain of Lys289 and the fumarate moiety of the substrate.

δ-Crystallin became unstable when the inter-subunit interaction that is provided by E294 was disrupted. Monitoring α-helix variation with increasing temperature, the mid-point temperature transition (*T*_m_) for E294L was about five degrees lower than for wild-type protein (Figure 2A and Table 1). Side-chain substitution in E294 apparently led to a destabilized secondary structure of δ-crystallin as judged by the thermal unfolding process. In the presence of Gdm-HCl, E294L underwent a similar multi-step unfolding process to that observed in the wild-type enzyme, as measured by tryptophan fluorescence (Figure 2B and Table 2). This unfolding process has been interpreted as dissociation of tetramers and then unfolding of monomers [17,18]. The major differences in unfolding transition between E294L and wild-type protein were at the first step (about 0 to 1 M Gdm-HCl), which is assumed to be the dimer dissociation step. The half Gdm-HCl concentration required (C_m_) for unfolding transition of E294L at this step was about 0.5 M, which is lower than that required for the wild-type protein (Table 2). Meanwhile, the total free energy required to unfold this mutant protein was about 7.5 kcal/mole decreased. This result demonstrates that L294 substitution acts to destabilize the quaternary structure of δ-crystallin. Disrupted interactions between dimer interfaces led to disassembly and the subsequent aggregation of dissociated dimers. A previous study in which the protruding N-terminal segment in goose δ-crystallin was truncated also resulted in dissociation of dimers from the tetramer and the subsequent formation of off-pathway aggregates [15]. This result implicates the NH_2_-terminus in the formation of the native quaternary structure, which requires proper alignment of the interfaces of the two dimers before association can occur. The inter-facial interactions that are provided by K315 and E327 are important. Disruption of the hydrogen bonds by side-chain substitution of these residues resulted in disassembly of the double dimer [19]. E294 is affected similarly because its side-chain provides interactions between dimer-dimer interfaces. However, our study shows that this interaction is critical for maintaining a hydrogen bonds network, which is required for catalysis, although not for the assembly of quaternary structure. Disruption of this interaction in δ-crystallin resulted in a mutant protein that had a similar conformation and quaternary structure to wild-type protein. However, the mutant protein was more susceptible to disassembly on exposure to denaturing conditions. This result indicates that the inherent stability is important in maintaining the structural integrity of lens proteins [28,29].

**Table 2:** Thermodynamic parameters of goose δ-crystallin unfolded in Gdm-HCl

**Proteins**	**ΔG_1_^o^**	**ΔG_2_^o^**	**ΔG_3_^o^**	**ΔG_t_^o^**	**[D]^1^_1/2_**	**[D]^2^_1/2_**	**[D]^3^_1/2_**
	**(kcal/mol)**				**(M)**		
WT	7.7±0.8	3.4±0.8	8.2±1.8	19.3	1.0±0.1	2.1±0.5	4.1±0.8
E294L	5.7±0.5	1.8±0.5	4.3±0.7	11.8	0.5±0.1	1.5±0.4	3.9±0.6

[D]_1/2_ is the half concentration of Gdm-HCl required in the unfolding transition of δ–crystallin. ΔG_t_^o^ is the summation of ΔG_1_^o^, ΔG_2_^o^, and ΔG_3_^o^. These values were derived from Figure 2B after global fitting the unfolding curve to the equation based on a 4-state model [15].
